# A wireless, implantable optoelectrochemical probe for optogenetic stimulation and dopamine detection

**DOI:** 10.1038/s41378-020-0176-9

**Published:** 2020-08-24

**Authors:** Changbo Liu, Yu Zhao, Xue Cai, Yang Xie, Taoyi Wang, Dali Cheng, Lizhu Li, Rongfeng Li, Yuping Deng, He Ding, Guoqing Lv, Guanlei Zhao, Lei Liu, Guisheng Zou, Meixin Feng, Qian Sun, Lan Yin, Xing Sheng

**Affiliations:** 1 School of Materials Science and Engineering and Hangzhou Innovation Institute, Beihang University, Beijing, 100191 China; 2 Department of Electronic Engineering, Beijing National Research Center for Information Science and Technology and IDG/McGovern Institute for Brain Research, Tsinghua University, Beijing, 100084 China; 3 Department of Physics, Tsinghua University, Beijing, 100084 China; 4 Beijing Institute of Collaborative Innovation, Beijing, 100094 China; 5 School of Materials Science and Engineering, Tsinghua University, Beijing, 100084 China; 6 Beijing Engineering Research Center of Mixed Reality and Advanced Display, School of Optics and Photonics, Beijing Institute of Technology, Beijing, 100081 China; 7 Department of Mechanical Engineering, Tsinghua University, Beijing, 100084 China; 8 Key Laboratory of Nano-devices and Applications, Suzhou Institute of Nano-Tech and Nano-Bionics, Chinese Academy of Sciences (CAS), Suzhou, 215123 China

**Keywords:** Electrical and electronic engineering, Biosensors

## Abstract

Physical and chemical technologies have been continuously progressing advances in neuroscience research. The development of research tools for closed-loop control and monitoring neural activities in behaving animals is highly desirable. In this paper, we introduce a wirelessly operated, miniaturized microprobe system for optical interrogation and neurochemical sensing in the deep brain. Via epitaxial liftoff and transfer printing, microscale light-emitting diodes (micro-LEDs) as light sources and poly(3,4-ethylenedioxythiophene) polystyrene sulfonate (PEDOT:PSS)-coated diamond films as electrochemical sensors are vertically assembled to form implantable optoelectrochemical probes for real-time optogenetic stimulation and dopamine detection capabilities. A customized, lightweight circuit module is employed for untethered, remote signal control, and data acquisition. After the probe is injected into the ventral tegmental area (VTA) of freely behaving mice, in vivo experiments clearly demonstrate the utilities of the multifunctional optoelectrochemical microprobe system for optogenetic interference of place preferences and detection of dopamine release. The presented options for material and device integrations provide a practical route to simultaneous optical control and electrochemical sensing of complex nervous systems.

## Optogenetics: Simultaneous sensing and control

A novel probe combines electrochemical sensing and optical control in a single wireless device, enabling researchers to remotely influence animal behavior and simultaneously measure their dopamine response. In optogenetic systems, light is used to switch on specific genetically-engineered neurons. A team led by Xing Sheng of China’s Tsinghua University engineered a device with a microscale LED for optogenetic stimulation and an electrochemical sensor to detect dopamine. The two elements are separated by a diamond film for electrical and thermal insulation. The resulting device is roughly 2.2 × 1.3 cm and weighs 2 grams, including a miniaturized wireless circuit for control and a rechargeable battery for power. The team used the device to remotely train mice to prefer one chamber of a test environment via optogenetic stimulation and to measure animals’ dopamine response.

## Introduction

In recent decades, the study of modern neuroscience has significantly advanced, along with the monumental progress of interdisciplinary technological innovations in fields, including electrophysiology^1^, optical interrogation/detection^2^ and neuropharmacology^3^. Although traditional electrical tools for neural recording and medication provide powerful capabilities for brain science research and clinical therapies of neurological disorders such as Parkinson’s disease and epilepsy^4^, the lack of cell-type specificity and side effects of stimulating untargeted brain regions impede their broader applications^5^. Recently developed genetically encoded optical actuators and indicators overcome the above limitations and offer more precise and cell-specific neural signal regulation and monitoring^6,7^. In addition, multifunctional optical neural interfaces, comprising highly integrated, implantable microscale optoelectronic, and microfluidic devices, have achieved remarkable accomplishments in optical/electrical/chemical multimodal sensing and modulation in the deep brain^8–12^. Representative examples include our recently developed optoelectronic probes for wireless optogenetic stimulation and fluorescence recording in freely moving rodents^13–17^. In addition to electrical pulses and calcium flows, neurotransmitters, which comprise a plethora of chemicals including dopamine, glutamate, serotonin, etc., are of critical importance and direct relevance in neural activities and brain functions^18,19^. Among the family of neurotransmitters, dopamine arouses particular interest because of its tight association with important behaviors such as motivation, reward, and reinforcement^20^. For example, morphological and neurochemical studies have noted prominent reductions in dopamine in the striatum of patients with Parkinson’s disease, and identifying the relationship between dopamine release and neuron stimulation is crucial for understanding the disease cause and developing precise treatments^21,22^. Therefore, it is highly desirable to develop a fully integrated implantable system leveraging optogenetic stimulators and dopamine sensors for closed-loop neural stimulation and recording. A commonly used method for dopamine detection involves tethered electrochemical probes made of carbon or noble metals such as gold and platinum, which could potentially be bundled with optical fibers for optical simulation^23–25^. More recently, genetically encoded fluorescence indicators of various neurotransmitters have also been actively exploited^26–29^. Integrating the detection of neurotransmitter levels within wirelessly operated optogenetic stimulation platforms in living animals, however, is relatively underexplored in state-of-the-art studies.

In this paper, we develop a wireless thin-film-based implantable microprobe system for optogenetic stimulation and electrochemical sensing of dopamine in the animal deep brain. A thin-film, microscale light-emitting diode (micro-LED) transferred on a flexible substrate is employed as a light source for optogenetic stimulation. A poly(3,4-ethylenedioxythiophene) polystyrene sulfonate (PEDOT:PSS)-coated diamond film is placed on the micro-LED, serving as an electrochemical sensor for dopamine detection. As an optically transparent, electrically insulating and thermally conductive interlayer in the stacked device structure, the diamond film separates the micro-LED and the PEDOT:PSS sensor, ensuring simultaneous optical stimulation and electrochemical detection in the same region. For wireless control and signal acquisition, the formed thin-film and flexible microprobe is interfaced with a miniaturized, removable circuit module powered by a rechargeable battery. Implanted into the ventral tegmental area (VTA) of freely behaving mice, the fully integrated optoelectrochemical microprobe is demonstrated to remotely control animal behaviors and dopamine signal recording.

## Results and discussion

### Device structure, fabrication, and functionality

**Fig. 1:**
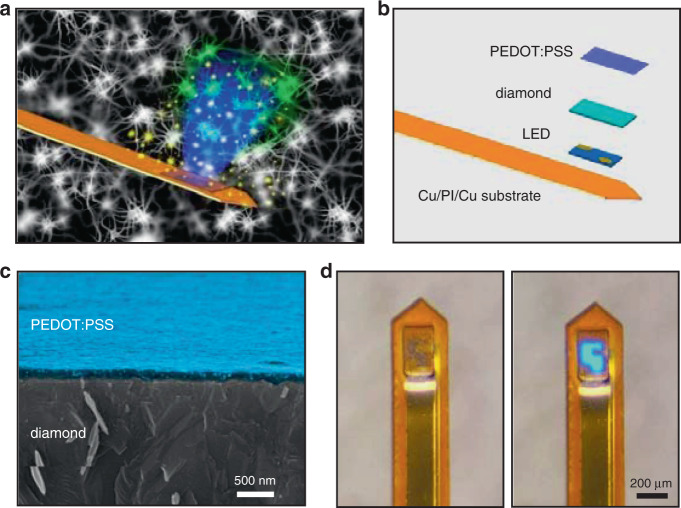
Structures of the implantable microprobe. **a** Conceptual illustration of the microprobe system for optogenetic stimulation and dopamine detection. **b** Exploded schematic of the microprobe, comprising a blue micro-LED, a diamond interlayer and a PEDOT:PSS thin film on the copper-coated polyimide (Cu/PI/Cu) substrate. **c** Cross-sectional scanning electron microscope (SEM) image of a PEDOT:PSS film (colorized in blue) coated on diamond. **d** Optical photographs of a fully fabricated microprobe (left: LED is off; right: LED is on, with an injection current of 0.1 mA)

Figure 1a schematically displays the conceptual illustration of our proposed implantable, fully integrated microprobe system for simultaneous optogenetic stimulation and electrochemical sensing in deep brain tissue, with an exploded view shown in Fig. 1b. Details about the device structure and fabrication processes are provided in the Supplementary Information (device fabrication and Supplementary Figs. S1–S3). The thin-film device stack incorporates a flexible, double-sided copper (Cu)-coated polyimide (PI) (18-μm Cu/25-μm PI/18-μm Cu) substrate, an indium gallium nitride (InGaN)-based blue-emitting micro-LED (size: 125 μm × 185 μm × 7 μm), an undoped diamond interlayer (size: 180 μm × 240 μm × 20 μm) and a PEDOT:PSS film (size: 150 μm × 200 μm × 0.1 μm). Here, we employ a Cu-coated PI film instead of pure PI as the supporting substrate because of the desirable heat dissipation of Cu films (Supplementary Fig. S4). The emission from the blue LED is utilized to optically control specific cells expressing light-sensitive ion channels, for example, channelrhodopsin-2 (ChR2)^6,7,30–32^, thereby activating or inhibiting neural activities. As an optically transparent and electrically conductive polymer layer, the PEDOT:PSS thin film can work as an effective electrochemical sensor for dopamine detection owing to its biocompatibility and low impedance at the biological interface^33–36^. As shown in Fig. 1b, the PEDOT:PSS film serves the working electrode (WE), while the reference electrode (RE) and the counter electrode (CE) are based on a standard Ag/AgCl wire and a platinum wire, respectively, which are integrated separately to reduce the device footprint. The optical transmittance of the PEDOT:PSS film is measured to be more than 90% in the visible spectral range (Supplementary Fig. S5). As an interlayer applied between the LED and the PEDOT:PSS sensor, the diamond film has manifold benefits: (1) Its optical transparency (>80%)^37^ ensures that the blue light can transmit through it and reach the bio-tissue; (2) its high thermal conductivity (>2000 W/m/K)^38^ allows for efficient heat dissipation during LED operation; (3) it electrically isolates the electrodes of the LED and the PEDOT:PSS film, minimizing signal cross talk; and (4) it could act as a liquid barrier and prevent the LED from failure in the aqueous environment. In addition, we note that the lateral dimensions of the diamond film are designed to be slightly larger than the micro-LED in order to (1) facilitate heat dissipation, (2) provide better encapsulation on the LED, and (3) improve the misalignment tolerance during the transfer and lithography processes. The LED is fabricated on sapphire, and the diamond film is grown on silicon; they are released from native substrates by laser liftoff and wet etching, forming freestanding films transferred onto PI using polydimethylsiloxane (PDMS) stamping methods^39,40^. On the diamond layer, the PEDOT:PSS film is conformally coated via spin casting and is lithographically patterned, with a thickness of ~100 nm (Fig. 1c). The micro-LED and the PEDOT:PSS film are metalized with sputter-coated Cr/Cu/Au and Cr/Au electrodes, respectively, while epoxy-based photoresist films (SU-8) serve as the bonding layer between the LED and the diamond, as well as the protective coating on metal electrodes. Finally, the flexible substrate is milled by an ultraviolet laser to realize a needle shape (width ~360 μm, length ~5 mm, shown in Fig. 1d), which is ready for implantation into the deep tissue of animal brains. Compared with other possible device geometries such as physically bonded fibers/wires^41,42^ or laterally placed thin-film sensors^16,43,44^, the vertically stacked device structure is more advantageous because (1) it has a higher spatial precision in the control and monitoring of a specifically targeted nucleus, which typically has sizes from tens to hundreds of micrometers in the mouse brain; and (2) such a thin-film geometry can have a smaller footprint and desirable mechanical flexibility^45^, significantly reducing tissue lesions and associated damage.

### Optoelectronic and thermal properties

**Fig. 2:**
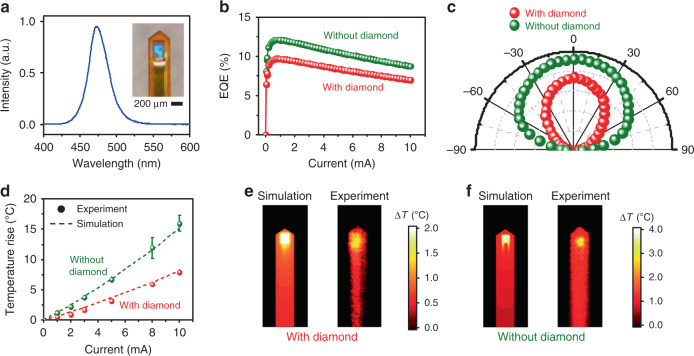
Optoelectronic and thermal characteritics of LEDs in the microprobe. **a** Electroluminescence spectrum of the InGaN blue LED. Inset: optical photograph of the microprobe. **b** External quantum efficiencies (EQEs) for LEDs with and without diamond/PEDOT:PSS coatings, under different injection currents. **c** Angular distribution of optical emissions for LEDs with and without diamond/PEDOT:PSS coatings. **d** Measured (dots) and simulated (dashed lines) maximum temperature rises above room temperature on the top surface of the LED probes (with and without diamond/PEDOT:PSS coatings) as a function of pulsed currents (frequency 20 Hz and duty cycle 20%). **e**, **f** Simulated (left) and measured (right) temperature distributions the LED probes (current 3 mA, frequency 20 Hz and duty cycle 20%), **e** with and **f** without diamond/PEDOT:PSS coatings

The utility of freestanding InGaN-based micro-LEDs for optogenetic stimulation has been demonstrated in previous works^40^. Here, we evaluate the optical and thermal properties of the micro-LED before and after integration with the PEDOT:PSS-coated diamond film for electrochemical sensing, with the results depicted in Fig. 2. Under current injection, the peak wavelength for the LED emission is approximately 470 nm (Fig. 2a), which matches the optical absorption of commonly used opsins such as ChR2 as optogenetic tools^15,30–32^. The external quantum efficiency (EQE) for LEDs with and without diamond and PEDOT:PSS films are plotted in Fig. 2b. The maximum EQEs are ~12% (for the bare LED) and ~10% (for the LED coated with diamond and PEDOT:PSS), obtained at an injection current of ~1 mA. In addition, both devices exhibit nearly Lambertian emission distributions (Fig. 2c). These results indicate that the diamond/PEDOT:PSS film placed on top of the blue LED does not greatly alter its emission characteristics, and the slight performance degradation (~20%) is in accordance with the high optical transmission of the diamond and PEDOT:PSS materials (>80%)^37,46^.

In addition to optical transparency, another prominent feature of the diamond film is its ultrahigh thermal conductivity. Even though the diamond film that we apply here has a polycrystalline morphology, its thermal conductivity still reaches as high as 2200 W/m/K, almost five times higher than that of copper films and three orders of magnitude higher than that of organic coatings, such as SU-8 and PDMS^47^. Such a thermally conductive coating is advantageous for heat dissipation of LEDs operated in biological systems. As shown in Fig. 2d–f, we evaluate the thermal behaviors of LEDs with and without diamond/PEDOT:PSS coatings. The LEDs are operated in ambient air under pulsed current injection (varied from 0 to 10 mA), with 20 Hz frequency and 20% duty cycle. Temperature maps are measured with an infrared camera and compared with thermal modeling established by finite element analysis. As shown in Fig. 2d, the maximum temperature increase for the LED with diamond is approximately half of that for the bare LED under the same injection currents. The thermal mapping results for LEDs operated at 3 mA are further depicted in Fig. 2e, f. These experimental results are in good agreement with the numerical predictions. At 3 mA, the probe surface temperature increase is restricted within 2 °C for the LED with the diamond coating, while the result for the bare LED is more than 4 °C. Furthermore, it should be noted that for the microprobe implanted in biological tissues, the increases in temperature, albeit more challenging to measure precisely, should be even lower than the results obtained in air because of the additional heat dissipation capability of biological tissues and fluids. The simulation results based on the established models are provided in Supplementary Fig. S6. This benefit is particularly favorable for optogenetic probes implanted in the deep brain since the mitigation of unwanted abnormal activities and possible tissue damage by overheating is critical for neurons^48^.

### Electrochemical characterization

#### The electrochemical properties of the PEDOT

**Fig. 3:**
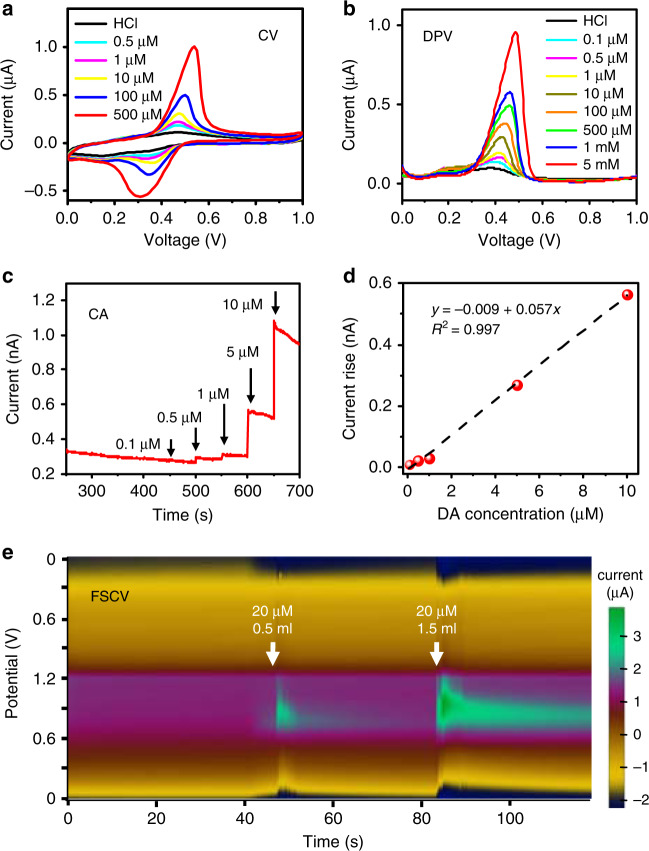
Electrochemical characteristics of the PEDOT:PSS film in the microprobe. **a** Cyclic voltammetry (CV). **b** Differential pulse voltammetry (DPV). **c** Chronoamperometry (CA). **d** Calibration curve: linear relationship between response currents and dopamine (DA) concentrations (0.1–10 μM). **e** Fast scan cyclic voltammetry (FSCV). All the experiments are performed in HCl solutions (pH = 4.0) with varied dopamine concentrations at room temperature, with a saturated Ag/AgCl electrode as the reference and a Pt sheet as the counter electrode

PSS electrode for dopamine sensing are studied and presented in Fig. 3. Although the normal dopamine level in the whole animal brain or other body parts is as low as a few pM^49,50^, it can be much higher (from several nM to several μM) in particular brain regions with large collections of dopaminergic neurons^51,52^. The detection of dopamine in aqueous solutions and biological environments is based on its redox reaction, in which dopamine is oxidized and converted into dopamine-o-quinone on the electrode surface, generating electric currents^53^. Cyclic voltammetry (CV), differential pulse voltammetry (DPV), chronoamperometry (CA), and fast scan cyclic voltammetry (FSCV) techniques are utilized to analyze the electrochemical features of the PEDOT:PSS electrode using a standard electrochemical workstation. A standard three-electrode configuration is applied, with the PEDOT:PSS film serving as the WE, silver/silver chloride (Ag/AgCl) and platinum (Pt) as the RE and the CE, respectively. Here, we use dilute hydrochloric acid (HCl) solutions (pH = 4.0) as solvents for dopamine in order to mitigate its degradation due to natural oxidation in air that causes measurement inaccuracy^54,55^. The dopamine concentrations used in the CV tests were 0 μM, 0.5 μM, 1 μM, 10 μM, 50 μM, 100 μM, and 500 μM. As shown in Fig. 3a, pairs of well-defined quasi-reversible redox peaks are observed in the presence of dopamine in HCl, with oxidation potentials of 0.5–0.6 V and the corresponding reduction potentials of 0.3–0.4 V. The peak redox currents increase with the concentration of dopamine in the solution. The DPV measurements in Fig. 3b reveal similar trends, with a higher sensitivity to the concentration and a better voltage resolution than the results obtained with CV analysis. Dopamine levels in HCl solutions as low as ~0.1 μM can be detected using DPV, and the detection limits using CV and CA are ~0.5 μM. Repetitive CV scanning curves of a representative probe are shown in Supplementary Fig. S7, demonstrating the reliability and repeatability of its electrochemical properties. DPV curves for multiple probes are also presented (Supplementary Fig. S8), in which the response differences among various probes are ascribed to the variations in the fabrication process (spin coating, misalignments in lithography and etching, etc.), as well as the instability of dopamine in the ambient environment. The dynamic current response at various dopamine concentrations is further determined using CA measurements, as shown in Fig. 3c, d. At a fixed bias voltage of 0.6 V, oxidation currents are captured as we change dopamine concentrations. In the range of 0.1–10 μM, a linear relationship between the dopamine concentration and the response current can be obtained, and the detection sensitivity is determined to be ~0.06 nA/μM. For our spin-coated PEDOT:PSS film with a working area of 150 μm × 200 μm defined by lithography, the normalized current response of dopamine by area is calculated to be 200 nA/μM/cm^2^, which is comparable with that measured by electrochemical sensors based on other materials^56,57^. In Fig. 3e, we further record the FSCV responses by dropping dopamine solutions in HCl, plotting the current response as a function of both time and voltage. It should be noted that CV, DPV, CA, and FSCV measurements exhibit different current sensitivities at different dopamine concentrations because part of the measured electrochemical currents is associated with the capacity at the electrode/electrolyte interface, which is highly sensitive to the application of different potential modulation forms and scan rates^58^. The experimental results performed in vitro clearly demonstrate the utility of applying the PEDOT:PSS film for real-time dopamine monitoring in biological systems.

### Circuit design and evaluation

**Fig. 4:**
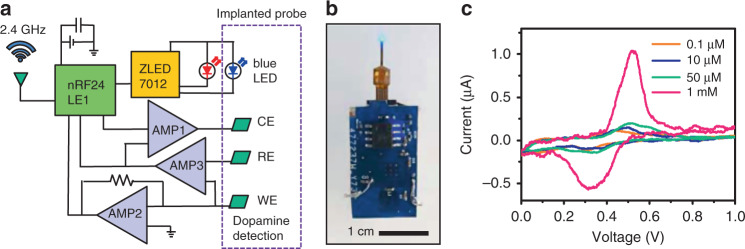
Circuit module for wireless operation. **a** Schematic diagram of the circuit. **b** Photograph of a circuit connected to an implantable probe with the micro-LED on. **c** CV curves acquired by the wireless circuit connect to a microprobe in HCl solutions with different dopamine levels

A customized circuit module is designed to wirelessly operate the implantable microprobe for integrated optoelectrochemical stimulation and sensing functionalities. The circuit diagram is depicted in Fig. 4a, with detailed layouts presented in Supplementary Figs. S9 and S10. Remote data communication is realized with a micro-controller (nRF24LE1, Nordic Semiconductor) with a transceiver operated at 2.4 GHz. A driver chip (ZLED7012, Renesas Electronics Corp.) provides a constant current to the micro-LED for optogenetic stimulation, with programmable current levels, frequencies, pulse widths, etc. A red LED is printed on the circuit outside the brain for signal indication. A digital-analogue converter (DAC60508, Texas Instruments) and two pre-amplifiers (OPA2381, Texas Instruments and ADA4505, Analog Devices Inc.) are connected to the chemical electrodes (WE, RE, and CE) for voltage scanning and current readout, achieving a resolution as low as 0.1 nA for current measurements. The analogue signals from the electrodes are collected, filtered, compared, and amplified in the operational network and sent to the corresponding ports of the micro-controller. After further data processing via the built-in program, the signals are wirelessly transmitted to the receiving end connected to a laptop computer via an antenna operated at a radio frequency of 2.4 GHz (Supplementary Fig. S11). The system is powered by a rechargeable lithium-ion battery with a capacity of 35 mAh. As shown in Fig. 4b, the microprobe consisting of a blue micro-LED and a PEDOT:PSS-coated diamond film as an electrochemical sensor is connected to the wireless circuit module with a footprint of ~2.2 cm × 1.3 cm and a weight of 2.0 g (including a 0.9-g battery). Via a flexible printed circuit connector, the manufactured circuit can be plugged into the implantable microprobe during in vivo experiments and detached from it when unnecessary. We evaluate the performance of our designed wireless circuit in vitro by taking CV scans in HCl solutions with various dopamine concentrations using a microprobe containing the PEDOT:PSS thin-film electrode. The measured CV results in Fig. 4c match reasonably well with those obtained using a commercial electrochemical analyzer, with clearly resolved redox peaks of dopamine. Similarly, CA measurements (current versus time at a fixed bias voltage) can also be performed. These results demonstrate the utility of the wireless circuit for in vivo tests in behaving animals.

### In vivo experiments

**Fig. 5:**
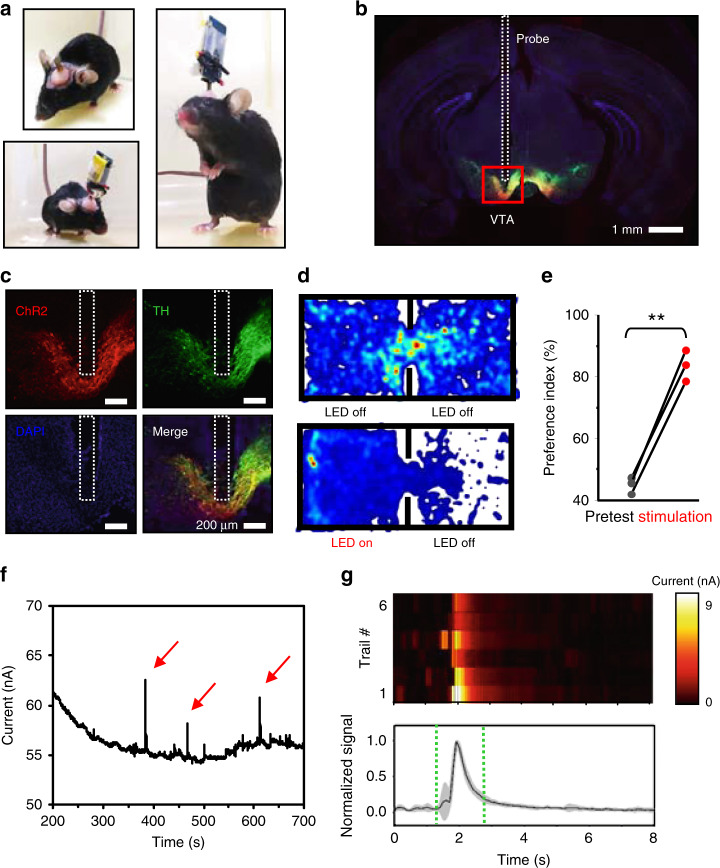
In vivo optogenetic stimulation and dopamine detection. **a** Photographs of an experimental mouse implanted with the microprobe, with and without the circuit module. **b** Immunostained fluorescence image of the brain section. **c** Immunostaining results of the VTA region expressing ChR2 (red), TH (green), DAPI (blue), as well as the merged image. **d** Position heat maps of the animal activity during pretests (top) and under optogenetic stimulations (bottom). Hotter colors represent longer duration in the location. **e** Preference indices (the ratio of the time that mice spend in the left chamber to the whole recorded time) on the left side under optogenetic stimulations, in comparison with those during pretests (*n* = 3 mice, unpaired *t* test, *P* < 0.01). Stimulation parameters: duration 10 ms, frequency 20 Hz, driving current 1.8 mA). **f** A representative amperometry curve (current versus time) measured with the PEDOT:PSS electrode in vivo. **g** Analyses of representative signal peaks. Top: Heat maps showing six individual trials (current signals vs. time) from two mice. Bottom: Average of normalized current signals

We further verify the performance of the wirelessly operated microprobe with in vivo optogenetic stimulation and dopamine sensing in experimental mouse models. The system can be easily mounted on the head of an adult mouse without affecting its behavior (Fig. 5a). Illustrated in Fig. 5b, probes including micro-LEDs and PEDOT:PSS electrodes are implanted into the ventral tegmental area (VTA), which is the origin of dopaminergic cells and involves reward circuitry^59,60^. Previous studies have shown that dopamine concentrations in the VTA typically range from several nM to several μM, depending on local neural activities^51,52^. A microprobe with a length of ~5 mm is implanted into the VTA of adult mice (DAT-Cre transgenic, 7–16 weeks) expressing ChR2, with detailed surgery procedures provided in Supplementary Figs. S12 and S13. Figure 5b shows a brain section with the lesion area created by the implanted microprobe. Details of the immunostained region in the VTA are provided in Fig. 5c, including stained ChR2 (red), rabbit polyclonal anti-tyrosine hydroxylase (TH, green), 4’,6-diamidino-2-phenylindole (DAPI, blue), and merged images. The track of the probe is indicated by the dashed boxes. The tissue damage and inflammatory responses are similar to those induced by the insertion of microprobes with similar geometries and flexible materials reported previously^16,61^. We perform real-time place preference (RTPP) tests by optogenetic stimulation 5 days after implanting microprobes into the VTA of mice. The mice are placed in a behavior box (50 cm × 25 cm × 25 cm) with two chambers and a gate with a width of 9 cm. Animals are allowed to explore the environment for 15 min to obtain a pretest result, and only those with no obvious place preference (Fig. 5d, upper part) are used for the optogenetic test. Recorded position heat maps and traces (Fig. 5d, as well as more raw data provided in Supplementary Fig. S14) clearly show that the implanted microprobe and the mounted wireless module impose few constraints on the animals’ locomotion. After the pretest, optical stimulation (duration: 10 ms, frequency: 20 Hz, driving current: 1.8 mA) generated by the blue-emitting micro-LED is provided for 15 min when mice enter the left chamber. The position heatmap (Fig. 5d, lower part) reveals that the mice exhibit obvious place preferences during the optogenetic stimulation period. The statistical results clearly show that animals spend more time in the stimulated chamber and have obvious place preferences compared with the situation in the pretests, indicating that dopaminergic neurons in the VTA are excited and that dopamine release is promoted. Optogenetic stimulation can be effective more than two weeks after micro-LED implantation (Supplementary Fig. S15). In fact, the implanted micro-LEDs and ChR2 expression can be stable for months after surgery, as previously demonstrated^14^.

To evaluate the electrochemical sensing capability, we measured CA curves (current versus time) with the PEDOT:PSS electrode implanted in the VTA. The VTA contains a mixture of dopaminergic (~65%), GABAergic (~30%), and glutamatergic (~5%) neurons^62^. Both gamma aminobutyric acid (GABA) and glutamate are non-electroactive^63,64^, thus making dopamine the dominant neurotransmitter probed in electrochemical tests in the VTA. Similar to the in vitro experiments shown in Fig. 3, we employ a Ag/AgCl wire and a stainless-steel screw as the reference and counter electrodes, respectively, which are implanted elsewhere in the mouse brain cortex. Here, the stainless steel screw is a standard fixture for the implantation surgery, so we directly use it as the counter electrode so that we do not have to implant another platinum wire, which would cause additional tissue damage. A constant forward bias of 0.6 V is applied between the WE and the RE, and the oxidation current flowing into the WE is acquired at an interval of 0.1 s. Figure 5f plots representative amperometric results measured with a microprobe implanted into the VTA of mice. Spontaneous current spikes are observed, and more signal traces from multiple animals are collected and analyzed in Fig. 5g (with raw data shown in Supplementary Fig. S16). The data collected from multiple individual trials reveal considerable consistency, with time constants matched to dopamine signals reported in other studies^65–67^. Here, we also observe that the microprobe can only be properly functional under acute tests in vivo, and its electrochemical sensitivity gradually degrades several hours after implantation because of potential chemicals and bio-substances adhering to the probe surface (as an example shown in Supplementary Fig. S17). Similar electrode fouling effects have also been reported for other electrochemical sensors based on, for example, carbon fibers^68^. As a future research thrust, this challenge needs to be addressed to enable chronic electrochemical detection in vivo.

## Conclusion

In summary, we report a wirelessly operated microprobe system for neural interrogation and neurotransmitter monitoring in the deep brain. Ultraminiaturized, vertically stacked micro-LED, diamond and PEDOT:PSS films are combined to realize optogenetic stimulation and dopamine sensing in a minimally invasive platform. The unique electrical, optical, and thermal properties of the PEDOT:PSS-coated diamond film make the device suitable for highly sensitive electrochemical sensing without affecting the micro-LED operation. A lightweight, remotely controlled circuit allows for behavioral studies on freely moving mice. In the future, more sophisticated in vivo experiments can be performed to demonstrate closed-loop operations, for example, modulating light stimulation in response to changes in dopamine levels or monitoring dopamine release when varying optical emissions. Endogenous dopamine release associated with various reward behaviors can also be probed. Moreover, the device capability can be further evaluated in animals with relevant disease models such as Parkinson’s disease and depression^61,69^, in which optogenetic stimulation can be utilized to activate or inhibit dopaminergic neurons to precisely control dopamine release in the brain. Advanced microfluidic devices can also be integrated into the platform for automated drug delivery, enabling pharmacological modulation of neural activities^11,12^. It should be noted that the sensing capability of the PEDOT:PSS electrodes may be interfered with by the micro-LED operation due to possible optoelectronic artefacts similar to those reported on other optotrodes^67,70^. In fact, we observed this kind of signal interference in experiments (Supplementary Fig. S18). To resolve these issues, strategies including modifying circuit design and incorporating new signal processing algorithms^71,72^ will be investigated in the future. In addition, multiple microprobes can be implanted into different brain regions in one or multiple animals, exploring functional connectivities among distinct units within the nervous system. The sensitivity to the dopamine concentration can be further optimized by adjusting the properties of the PEDOT:PSS film, including its geometries, doping, surface treatment, etc. Considering that various neurotransmitters such as glutamate, serotonin, and norepinephrine exist in the neural system, the specificity and sensitivity of dopamine can be further improved by selective coatings including metal nanoparticles (Au^73,74^, Pt^75^, Pd^76^), enzymes (laccase^77^, tyrosinase^78,79^), and carbon-based materials^80,81^ on PEDOT:PSS^79^. For most electrochemical sensors used for in vivo experiments, trade-offs between sensitivity, selectivity and stability should be taken into account^70,82^. Although we only demonstrate acute tests in living mice, chronic operation of electrochemical sensors is also a key challenge for complex behavior experiments. In addition to optogenetics and electrochemistry, many research tools including fluorescence photometry^16^, electrophysiology^83^, and microfluidics^84^ can be combined in such a platform to accomplish multimodal electrical-optical-chemical closed-loop modulation and detection of neural activities. In addition, the circuit design can be further optimized, for example, by incorporating flexible circuit boards and battery-free energy-harvesting techniques^84–86^. Collectively, we envision that these advanced strategies provide a promising route to tackle unresolved challenges in fundamental neuroscience and medical practices.

## Materials and methods

### Device fabrication

Details about the device structures and fabrication processes are provided in the Supplementary Information (device fabrication and Supplementary Figs. S1–S3).

#### Micro-LEDs

Details for the micro-LED fabrication are described in the previous work^40^. The InGaN-based blue LEDs are epitaxially grown on sapphire substrates using metal–organic chemical vapor deposition (MOCVD) and are lithographically formed. The structure (from bottom to top) consisted of a GaN buffer layer, an n-GaN layer, an InGaN/GaN multiple-quantum-well layer, and a p-GaN layer, with a total thickness of approximately 7.1 μm. The lateral dimension of the LED is defined by inductively coupled plasma reactive ion etching (ICP-RIE), with 4 mTorr pressure, 40 sccm Cl_2_, 5 sccm BCl_3_, 5 sccm Ar, ICP power of 450 W, bias power of 75 W, and an etch rate of 0.33 μm/min. Freestanding thin-film micro-LEDs (dimension: 180 μm × 125 μm × 7 μm) are formed by laser liftoff (Light Source: the KrF excimer laser at 248 nm, Coherent, Inc., CompexPro110). The optimized power density for the laser is ~0.7 J/cm^2^.

#### Freestanding diamond film

The polycrystalline diamond film (thickness ~20 μm) is grown on single-crystalline silicon substrates via chemical vapor deposition (CVD). Laser milling (Nd:YVO4 laser, 1064 nm) is used to cut the film into designed patterns (size: 180 μm × 240 μm) and shapes. The silicon substrate is removed using an etchant solution (CH_3_COOH:HNO_3_: HF = 5:5:2 by volume ratio), realizing freestanding diamond films.

#### Fabrication of the microprobe

A 10-µm-thick polyimide is coated on the Cu/PI/Cu substrate and baked at 250 °C for over 120 min as an insulating layer between the micro-LED and the substrate. Using pre-patterned polydimethylsiloxane (PDMS) (Dow Corning Sylgard 184 kit, 1:10 weight ratio) stamps, the freestanding LEDs are transferred onto flexible double side Cu-coated polyimide (18-μm Cu/25-μm PI/18-μm Cu, from DuPont) substrates, with a spin-coated adhesive layer. LEDs are encapsulated with 5-μm-thick SU-8 (SU8-3005) as an insulating layer. Sputtered layers of 10-nm Cr/500-nm Cu/100-nm Au serve as the metalized electrode for the micro-LEDs, and the 5-μm-thick SU-8 coating (SU8-3005) serves as an encapsulation layer. The freestanding diamonds are transferred onto micro-LEDs using similar PDMS stamps with 2-μm-thick SU-8 (SU8-2002) as an adhesive layer. A 5-μm-thick SU-8 (SU8-3005) film is spin-coated on the sample, with the surface of the diamond lithographically exposed. Sputtered 5-nm Cr/500-nm Au films are used as interconnected electrodes for the PEDOT:PSS films. The PEDOT:PSS film is coated on the diamond as the working electrochemical electrode and is patterned by reactive ion etching (RIE) (90 mTorr pressure, 100 sccm O_2_, 5 sccm SF_6_, power 100 W for 200 s). Another photoresist film (SU8-3005, 5-μm thick) is patterned on the metal electrodes as a waterproof layer. Subsequently, the flexible substrates are patterned to form needle shapes (width ~360 μm, length ~5 mm) by UV laser milling.

### Device characterization

SEM images are captured by a ZEISS Merlin microscope (15 kV). Optical microscopy images are taken by an MC-D800U(C) microscope (Phenix Optics Co., Ltd.). The EQE of micro-LEDs are measured using an integrating sphere (Labsphere Inc.) and a calibrated Si photodetector. Thermal images are acquired by an infrared camera (FOTRIC 228). LED emission spectra are collected with a spectrometer (Ocean Optics HR2000 + ). Far-field angular emission profiles of micro-LEDs are captured by a standard Si photodetector (DET36A, Thorlabs). In vitro electrochemical tests of dopamine detection are performed with an electrochemical workstation (CHI 650E, Shanghai Chenhua Co., Ltd, China). CV is performed at a sweep rate of 0.1 V/s from 0 V to 1 V. The conditions for the DPV are as follows: voltage step, 4 mV; pulse time, 200 ms; pulse amplitude, 50 mV; and scan rate, 20 mV/s. CA is performed at a constant bias of 0.6 V. FSCV is performed with a sweep rate of 10 V/s from 0 V to 1.2 V for 2000 cycles. All of the electrochemical measurements are carried out in a Faraday cage to avoid electromagnetic disturbance from the environment.

#### Thermal simulation

The simulations of temperature increase on the surface of LED probes (with and without diamond) are performed by steady-state finite element methods in COMSOL Multiphysics®, using the module of Heat Transfer in Solids. The three-dimensional geometric model of the probed is constructed based on the structure described in this paper. The InGaN LED is defined as the heat source, with the input thermal power density (W/m^3^) estimated by Eq. (1)

1 $$Q = V \times I \times \left( {1-{\mathrm{EQE}}} \right) \times D/V_{{\mathrm{LED}}}$$

where *V* and *I* are the measured voltage and current of the LED under continuous current injection conditions, respectively, EQE is the external quantum efficiency, *D* is the duty cycle of the LED in the experiments (20% in this study) and *V*_LED_ is the volume of the LED. Boundary conditions of natural convection to air are applied to all external surfaces of the probe model, in accordance with the experimental setup, and the air temperature is set to 20 °C. The temperature distribution T is calculated by solving the following heat transfer Eqs. (2) and (3):

2 $$\rho C_p{\mathbf{u}} \cdot \nabla T + \nabla \cdot {\mathbf{q}} = Q$$

3 $${\mathbf{q}} = - \kappa \nabla T$$

where *ρ* is the material mass density, *C*_*p*_ is the heat capacity at constant pressure, and *κ* is the thermal conductivity. A self-adaptive mesh setup is applied on the geometric model. Solutions of temperature are acquired by a stationary fully coupled solver within the convergence tolerance of 10^–5^ using Newton iteration methods, and the final results of temperature changes are plotted on the external surface of the probe model from the top view.

### In vivo studies

#### Stereotaxic surgery

The animal protocols were approved by the Animal Care and Use Committee of Tsinghua University and the National Institute of Biological Sciences, Beijing. Adult (7–16 weeks) DAT-Cre transgenic mice (Jackson Laboratory, strain B6.SJL-Slc6a3tm1.1(cre)Bkmn/J) are used. Mice are group housed at a constant temperature (23 ± 1 °C) and in a 12-h light/12-h dark cycle (lights on 20:00–08:00). Adult DAT-Cre mice are anaesthetized by intraperitoneal injection of 1% pentobarbital sodium (60 mg/kg) and then placed in a stereotaxic instrument (68025, RWD). After sterilization of the site with 75% alcohol, incisions are made to expose the skull, and the skull is aligned to standard stereotaxic coordinates. In the skull, a small hole with a diameter of ~500 μm is formed by a drill, and then, 500 nl of virus (AAV2/9-hEF1a-DIO-hChR2(H134R)-mCherry-WPRE-pA) is injected at a rate of 46 nl/min by a glass micropipette using a microsyringe pump (Nanoliter 2000 injector with the Micro4 controller, WPI) into the VTA (stereotaxic coordinates from bregma (mm): anterior–posterior (AP): − 3.40, medial–lateral (ML): +/−0.50, dorsal–ventral (DV): − 4.20). After the wound is sutured, the mice recover with the virus expressing for 3 weeks, and a second surgery is performed to implant the three electrodes. The implantation procedure is similar to the injection procedure. After fixing the anaesthetized mouse in a stereotaxic instrument and exposing the skull, two holes are made by a drill. A stainless steel screw tied with a Ag wire is inserted into the brain to work as the counter electrode. The skull surface is dried, and then, the microprobe is implanted into the VTA (stereotaxic coordinates from bregma (mm): anterior–posterior (AP): − 3.40, medial–lateral (ML): +/−0.52, dorsal–ventral (DV): − 4.50). Dental cement is used to affix the probe and the stainless steel screw. A third hole is made in the skull after the dental cement is dried, then a Ag/AgCl wire is implanted to work as the reference electrode, and dental cement is used again to affix the three electrodes. Both the CE and RE are implanted elsewhere in the mouse brain cortex (Supplementary Fig. S12). For the implantation of the probe used for RTPP, photographs of the surgical procedure are provided (Supplementary Fig. S13).

#### Immunohistochemistry

Mice are perfused with phosphate-buffered saline (PBS) solution followed by 4% paraformaldehyde. Mouse brains are postfixed in the same paraformaldehyde solution overnight and then immersed in 30% sucrose solution for 24 h. The frozen brains are cut into sections with a thickness of 40 μm, and then, the sections are washed with PBS and PBST (0.3% Triton X-100 in PBS) and blocked in 3% bovine serum albumin (BSA) in PBST at room temperature for 1 h. The sections are incubated with primary antibodies (rabbit polyclonal anti-tyrosine hydroxylase antibody, 1:1000) for 24 h at 24 °C, washed with PBST 5 times, incubated with secondary antibodies (goat anti-rabbit IgG (H + L) cross-adsorbed secondary antibody, Alexa Fluor 488, 1:500) for 2 h at room temperature (avoiding light), and finally washed with PBS three times. Fluorescent images are scanned with an automated slider scanner (17026728, Zeiss) and a laser scanning microscope (FV3000, Olympus).

## Supplementary information

SUPPLEMENTAL MATERIAL

## Data Availability

All data needed to evaluate the conclusions in the paper are present in the paper and/or the Supplementary Materials. Additional data related to this paper may be requested from the authors.

## References

[1] Fox KCR, Foster BL, Kucyi A, Daitch AL, Parvizi J (2018). Intracranial electrophysiology of the human default network. Trends Cogn. Sci..

[2] Kim CK, Adhikari A, Deisseroth K (2017). Integration of optogenetics with complementary methodologies in systems neuroscience. Nat. Rev. Neurosci..

[3] Kamp F (2019). Effects of sedative drug use on the dopamine system: a systematic review and meta-analysis of in vivo neuroimaging studies. Neuropsychopharmacology.

[4] Cogan SF (2008). Neural stimulation and recording electrodes. Annu. Rev. Biomed. Eng..

[5] Aravanis AM (2007). An optical neural interface:in vivocontrol of rodent motor cortex with integrated fiberoptic and optogenetic technology. J. Neural Eng..

[6] Kampasi K (2018). Dual color optogenetic control of neural populations using low-noise, multishank optoelectrodes. Microsyst. Nanoengineering.

[7] Khan W (2019). Inductively coupled, mm-sized, single channel optical neuro-stimulator with intensity enhancer. Microsyst. Nanoeng..

[8] Pashaie R (2013). Optogenetic brain interfaces. IEEE Rev. Biomed. Eng..

[9] Park S (2017). One-step optogenetics with multifunctional flexible polymer fibers. Nat. Neurosci..

[10] Fan B (2016). A hybrid neural interface optrode with a polycrystalline diamond heat spreader for optogenetics. Technology.

[11] Jeong J-W (2015). Wireless optofluidic systems for programmable in vivo pharmacology and optogenetics. Cell.

[12] Qazi R (2019). Wireless optofluidic brain probes for chronic neuropharmacology and photostimulation. Nat. Biomed. Eng..

[13] Kim T-i (2013). Injectable, cellular-scale optoelectronics with applications for wireless optogenetics. Science.

[14] Shin G (2017). Flexible near-field wireless optoelectronics as subdermal implants for broad applications in optogenetics. Neuron.

[15] Zhao Y (2018). Wirelessly operated, implantable optoelectronic probes for optogenetics in freely moving animals. IEEE Trans. Electron Devices.

[16] Lu L (2018). Wireless optoelectronic photometers for monitoring neuronal dynamics in the deep brain. Proc. Natl Acad. Sci. USA.

[17] Ding H (2018). Microscale optoelectronic infrared-to-visible upconversion devices and their use as injectable light sources. Proc. Natl Acad. Sci. USA.

[18] Trimbuch T, Rosenmund C (2016). Should I stop or should I go? The role of complexin in neurotransmitter release. Nat. Rev. Neurosci..

[19] Johnson KVA, Foster KR (2018). Why does the microbiome affect behaviour?. Nat. Rev. Microbiol..

[20] Berke JD (2018). What does dopamine mean?. Nat. Neurosci..

[21] Jankovic J (2017). Movement disorders in 2016: progress in Parkinson disease and other movement disorders. Nat. Rev. Neurol..

[22] Zhao X-d (2009). Long term high frequency stimulation of STN increases dopamine in the corpus striatum of hemiparkinsonian rhesus monkey. Brain Res..

[23] Bass CE (2010). Optogenetic control of striatal dopamine release in rats. J. Neurochemistry.

[24] Chaudhury D (2013). Rapid regulation of depression-related behaviours by control of midbrain dopamine neurons. Nature.

[25] Tye KM (2013). Dopamine neurons modulate neural encoding and expression of depression-related behaviour. Nature.

[26] Jing M (2018). A genetically encoded fluorescent acetylcholine indicator for in vitro and in vivo studies. Nat. Biotechnol..

[27] Marvin JS (2019). A genetically encoded fluorescent sensor for in vivo imaging of GABA. Nat. Methods.

[28] Feng J (2019). A genetically encoded fluorescent sensor for rapid and specific in vivo detection of norepinephrine. Neuron.

[29] Sun F (2018). A genetically encoded fluorescent sensor enables rapid and specific detection of dopamine in flies, fish, and mice. Cell.

[30] Grossman N (2010). Multi-site optical excitation using ChR2 and micro-LED array. J. Neural Eng..

[31] Iwai Y, Honda S, Ozeki H, Hashimoto M, Hirase H (2011). A simple head-mountable LED device for chronic stimulation of optogenetic molecules in freely moving mice. Neurosci. Res..

[32] Poher V (2008). Micro-LED arrays: a tool for two-dimensional neuron stimulation. J. Phys. D: Appl. Phys..

[33] Vreeland RF (2015). Biocompatible PEDOT: nafion composite electrode coatings for selective detection of neurotransmitters in vivo. Anal. Chem..

[34] Xu G, Li B, Cui XT, Ling L, Luo X (2013). Electrodeposited conducting polymer PEDOT doped with pure carbon nanotubes for the detection of dopamine in the presence of ascorbic acid. Sens. Actuators B: Chem..

[35] Xu G, Jarjes ZA, Desprez V, Kilmartin PA, Travas-Sejdic J (2018). Sensitive, selective, disposable electrochemical dopamine sensor based on PEDOT-modified laser scribed graphene. Biosens. Bioelectron..

[36] Demuru S, Deligianni H (2017). Surface PEDOT: nafion coatings for enhanced dopamine, serotonin and adenosine sensing. J. Electrochem. Soc..

[37] Walker J (1979). Optical absorption and luminescence in diamond. Rep. Prog. Phys..

[38] Wei L, Kuo P, Thomas R, Anthony T, Banholzer W (1993). Thermal conductivity of isotopically modified single crystal diamond. Phys. Rev. Lett..

[39] Park S-I (2009). Printed assemblies of inorganic light-emitting diodes for deformable and semitransparent displays. science.

[40] Li L (2018). Heterogeneous integration of microscale GaN light‐emitting diodes and their electrical, optical, and thermal characteristics on flexible substrates. Adv. Mater. Technol..

[41] Witten IB (2011). Recombinase-driver rat lines: tools, techniques, and optogenetic application to dopamine-mediated reinforcement. Neuron.

[42] Stauffer WR (2016). Dopamine neuron-specific optogenetic stimulation in rhesus macaques. Cell.

[43] Kwon KY, Sirowatka B, Weber A, Li W (2013). Opto-μECoG array: a hybrid neural interface with transparent μECoG electrode array and integrated LEDs for optogenetics. IEEE Trans. Biomed. Circuits Syst..

[44] Yoshimoto, S. et al. Implantable wireless 64-channel system with flexible ECoG electrode and optogenetics probe. In *2016 IEEE Biomedical Circuits and Systems Conference (BioCAS)*, 476–479 (IEEE, 2016).

[45] Park SI (2015). Soft, stretchable, fully implantable miniaturized optoelectronic systems for wireless optogenetics. Nat. Biotechnol..

[46] Admassie S (2006). A polymer photodiode using vapour-phase polymerized PEDOT as an anode. Sol. Energy Mater. Sol. Cells.

[47] Hartmann J, Voigt P, Reichling M (1997). Measuring local thermal conductivity in polycrystalline diamond with a high resolution photothermal microscope. J. Appl. Phys..

[48] Owen SF, Liu MH, Kreitzer AC (2019). Thermal constraints on in vivo optogenetic manipulations. Nat. Neurosci..

[49] Labib M, Sargent EH, Kelley SO (2016). Electrochemical methods for the analysis of clinically relevant biomolecules. Chem. Rev..

[50] Peaston RT, Weinkove C (2004). Measurement of catecholamines and their metabolites. Ann. Clin. Biochem..

[51] Ferapontova EE (2017). Electrochemical analysis of dopamine: perspectives of specific in vivo detection. Electrochim. Acta.

[52] Robinson, D. L. & Wightman, R. M. Rapid dopamine release in freely moving rats. In Electrochemical Methods Neuroscience. (eds Michael, A. C. & Borland, L. M.) Ch. 2 (CRC Press, Taylor & Francis, 2007). 21204389

[53] Venton BJ, Wightman RM (2003). Psychoanalytical electrochemistry: dopamine and behavior. Anal. Chem..

[54] Thorré K, Pravda M, Sarre S, Ebinger G, Michotte Y (1997). New antioxidant mixture for long term stability of serotonin, dopamine and their metabolites in automated microbore liquid chromatography with dual electrochemical detection. J. Chromatogr. B: Biomed. Sci. Appl..

[55] Kankaanpää A, Meririnne E, Ariniemi K, Seppälä T (2001). Oxalic acid stabilizes dopamine, serotonin, and their metabolites in automated liquid chromatography with electrochemical detection. J. Chromatogr. B: Biomed. Sci. Appl..

[56] Taylor IM (2017). Enhanced dopamine detection sensitivity by PEDOT/graphene oxide coating on in vivo carbon fiber electrodes. Biosens. Bioelectron..

[57] Belaidi FS (2015). PEDOT-modified integrated microelectrodes for the detection of ascorbic acid, dopamine and uric acid. Sens. Actuators B: Chem..

[58] Scholz F (2015). Voltammetric techniques of analysis: the essentials. ChemTexts.

[59] Brischoux F, Chakraborty S, Brierley DI, Ungless MA (2009). Phasic excitation of dopamine neurons in ventral VTA by noxious stimuli. Proc. Natl Acad. Sci. USA.

[60] Beier KT (2015). Circuit architecture of VTA dopamine neurons revealed by systematic input-output mapping. Cell.

[61] Zhang Y (2019). Battery-free, lightweight, injectable microsystem for in vivo wireless pharmacology and optogenetics. Proc. Natl Acad. Sci. USA.

[62] van Zessen R, Phillips JL, Budygin EA, Stuber GD (2012). Activation of VTA GABA neurons disrupts reward consumption. Neuron.

[63] Hu Y, Mitchell KM, Albahadily FN, Michaelis EK, Wilson GS (1994). Direct measurement of glutamate release in the brain using a dual enzyme-based electrochemical sensor. Brain Res..

[64] Hossain I (2018). A novel microbiosensor microarray for continuous ex vivo monitoring of gamma-aminobutyric acid in real-time. Front. Neurosci..

[65] Lu Y, Driscoll N, Ozden I, Yu Z, Nurmikko AV (2015). Modulating dopamine release by optogenetics in transgenic mice reveals terminal dopaminergic dynamics. Neurophotonics.

[66] Bass CE, Grinevich VP, Kulikova AD, Bonin KD, Budygin EA (2013). Terminal effects of optogenetic stimulation on dopamine dynamics in rat striatum. J. Neurosci. Methods.

[67] Chiu W-T (2014). Real-Time Electrochemical Recording of Dopamine Release under Optogenetic Stimulation. PLoS ONE.

[68] Zhang M (2007). Carbon nanotube-modified carbon fiber microelectrodes for in vivo voltammetric measurement of ascorbic acid in rat brain. Anal. Chem..

[69] Zhang S (2018). Real-time simultaneous recording of electrophysiological activities and dopamine overflow in the deep brain nuclei of a non-human primate with Parkinson’s disease using nano-based microelectrode arrays. Microsyst. Nanoeng..

[70] Yang C, Denno ME, Pyakurel P, Venton BJ (2015). Recent trends in carbon nanomaterial-based electrochemical sensors for biomolecules: a review. Analytica Chim. Acta.

[71] Sun Y (2014). A novel method for removal of deep brain stimulation artifact from electroencephalography. J. Neurosci. Methods.

[72] Zamani, H., Bahrami, H., Garris, P. A. & Mohseni, P. On the use of compressive sensing (CS) for brain dopamine recording with fast-scan cyclic voltammetry (FSCV). In *2017 IEEE International Symposium on Circuits and Systems**(ISCAS)*, 1–4 (IEEE, 2017).

[73] Zhu Q (2017). 3D Graphene hydrogel–gold nanoparticles nanocomposite modified glassy carbon electrode for the simultaneous determination of ascorbic acid, dopamine and uric acid. Sens. Actuators B: Chem..

[74] Wei M (2008). Selective determination of dopamine on a boron-doped diamond electrode modified with gold nanoparticle/polyelectrolyte-coated polystyrene colloids. Adv. Funct. Mater..

[75] Sun C-L, Lee H-H, Yang J-M, Wu C-C (2011). The simultaneous electrochemical detection of ascorbic acid, dopamine, and uric acid using graphene/size-selected Pt nanocomposites. Biosens. Bioelectron..

[76] Jiang J, Du X (2014). Sensitive electrochemical sensors for simultaneous determination of ascorbic acid, dopamine, and uric acid based on Au@Pd-reduced graphene oxide nanocomposites. Nanoscale.

[77] Lin Y (2010). A non-oxidative electrochemical approach to online measurements of dopamine release through laccase-catalyzed oxidation and intramolecular cyclization of dopamine. Biosens. Bioelectron..

[78] Maciejewska J (2011). Selective detection of dopamine on poly(indole-5-carboxylic acid)/tyrosinase electrode. Electrochim. Acta.

[79] Njagi J, Chernov MM, Leiter J, Andreescu S (2010). Amperometric detection of dopamine in vivo with an enzyme based carbon fiber microbiosensor. Anal. Chem..

[80] Kumar S, Ahlawat W, Kumar R, Dilbaghi N (2015). Graphene, carbon nanotubes, zinc oxide and gold as elite nanomaterials for fabrication of biosensors for healthcare. Biosens. Bioelectron..

[81] Maiti D, Tong X, Mou X, Yang K (2019). Carbon-based nanomaterials for biomedical applications: a recent study. Front. Pharmacol.

[82] Venton BJ, Cao Q (2020). Fundamentals of fast-scan cyclic voltammetry for dopamine detection. Analyst.

[83] Patel AA, McAlinden N, Mathieson K, Sakata S (2020). Simultaneous Electrophysiology and Fiber Photometry in Freely Behaving Mice. Front. Neurosci.

[84] Bandodkar AJ (2019). Battery-free, skin-interfaced microfluidic/electronic systems for simultaneous electrochemical, colorimetric, and volumetric analysis of sweat. Sci. Adv..

[85] Kim J (2016). Battery-free, stretchable optoelectronic systems for wireless optical characterization of the skin. Sci. Adv..

[86] Han S (2018). Battery-free, wireless sensors for full-body pressure and temperature mapping. Sci. Transl. Med..

