# Supplementary material for: A wireless, implantable optoelectrochemical probe for optogenetic stimulation and dopamine detection
Source: Microsyst Nanoeng. 2020 Aug 24;6:64. doi: 10.1038/s41378-020-0176-9 (PMC8433152; doi:10.1038/s41378-020-0176-9)
Supplement: Supplementary file 1 — SUPPLEMENTAL MATERIAL [file 41378_2020_176_MOESM1_ESM.doc]

**Supplemental Information**

**A wireless,** **implantable optoelectrochemical probe for optogenetic stimulation and dopamine detection**

Changbo Liu,^1^ Yu Zhao,^2^ Xue Cai,^2^ Yang Xie,^2^ Taoyi Wang,^3^ Dali Cheng,^2^ Lizhu Li,^2^ Rongfeng Li,^4^ Yuping Deng,^5^ He Ding,^6^ Guoqing Lv,^6^ Guanlei Zhao,^7^ Lei Liu,^7^ Guisheng Zou,^7^ Meixin Feng,^8^ Qian Sun,^8^ Lan Yin,^5^ Xing Sheng^2, *^

**Affiliations**

^1^School of Materials Science and Engineering and Hangzhou Innovation Institute, Beihang University, Beijing, 100191, China

^2^Department of Electronic Engineering, Beijing National Research Center for Information Science and Technology and IDG/McGovern Institute for Brain Research, Tsinghua University, Beijing, 100084, China

^3^Department of Physics, Tsinghua University, Beijing, 100084, China

^4^Beijing Institute of Collaborative Innovation, Beijing, 100094, China

^5^School of Materials Science and Engineering, Tsinghua University, Beijing, 100084 China

^6^Beijing Engineering Research Center of Mixed Reality and Advanced Display, School of Optics and Photonics, Beijing Institute of Technology, Beijing, 100081, China

^7^Department of Mechanical Engineering, Tsinghua University, Beijing, 100084, China

^8^Key Laboratory of Nano-devices and Applications, Suzhou Institute of Nano-Tech and Nano-Bionics, Chinese Academy of Sciences (CAS), Suzhou 215123, China

*Corresponding author: Xing Sheng, [xingsheng@tsinghua.edu.cn](mailto:xingsheng@tsinghua.edu.cn)

Device Fabrication (Page 2–9)

Figures S1–S18

**Device fabrication**

The detailed structure of our proposed optoelectrochemical probe involves (from bottom to top): an flexible double side copper (Cu) coated polyimide (PI) (18 μm Cu / 25 μm PI / 18 μm Cu) substrate, an indium gallium nitride (InGaN) based blue emitting micro-LED (size: 125 μm × 185 μm × 7 μm), an undoped diamond interlayer (size: 180 μm × 240 μm × 20 μm) and a PEDOT:PSS film (size: 150 μm × 200 μm × 0.1 μm).

A detailed description of the process to fabricate the implantable, fully integrated microprobe system is listed below:

*LED Fabrication*

The LED structure (from bottom) included the sapphire substrate, a GaN buffer layer, an n-GaN, an InGaN/GaN multiple-quantum-well layer, and a p-GaN. The processes for LED fabrication are the same as those described our previous work.

Reference: Li, L. et al. Heterogeneous Integration of Microscale GaN Light-Emitting Diodes and Their Electrical, Optical, and Thermal Characteristics on Flexible Substrates. *Advanced Materials Technologies* **3**, 1700239 (2018).

*Preparation of the Adhesive Solution*

The adhesive solution comprises a mixture of:

bisphenol A glycerolate (1 glycerol/phenol) diacrylate;

3-(Trimethoxysilyl) propyl methacrylate;

Spin-on-Glass (SOG 500F, Filmtronics Inc.);

2-Benzyl-2-(dimethylamino)-4’-morpholinobutyrophenone;

and anhydrous ethanol.

The weight ratio is 200:100:100:9:2000. Stir at room temperature until full mixing. Store the mixed solution in refrigerator (4 °C) for future use.

Reference: Kim, T. et al. Thin Film Receiver Materials for Deterministic Assembly by Transfer Printing. *Chemstry of Materials* **26**, 3502 (2014).

*Diamond Fabrication*

1. The undoped diamond film is grown on a silicon substrate by chemical vapor deposition (CVD).
2. Clean the grown diamond with acetone, isopropyl alcohol (IPA), deinoized (DI) water.
3. Laser cutting (Nd:YVO_4_ laser, 1064 nm) with a power of 7 W, pulse repetition rate of 1 MHz, scan speed of 1 m/s and repeat scan for 200 times.
4. Etch the Si substrate in CH_3_COOH : HNO_3_ : HF = 5:5:2 by volume for 0.5 hour to release the diamond film, and rinse with DI water.

*PEDOT:PSS Preparation*

1. Clean a brown glass bottle with standard RCA clean process 1 (NH_4_OH : H_2_O_2_ : H_2_O = 1:1:5, 80 °C) for 10 min.
2. Fully mix 10 ml 100.00% PEDOT:PSS (CLEVIOS PH 1000, Xi'an Polymer Light Technology Corp.) with 0.5 ml of Ethylene glycol (AR, Beijing Lanyi chemical products Co., Ltd) , 10μL of Dodecylbenzene sulfonate (DBSA) (90%, Shanghai Aladdin Bio-Chem Technology Co., LTD) and 0.1ml 3-Glycidoxypropyltrimethoxysilane (GOPS) (97%, J&K Scientific Ltd.) for 1 h.
3. Store the mixed solution in refrigerator (4 °C) for future use.

*LED transfer printing*

1. Clean a glass substrate with standard RCA clean process 1 for 10 min.
2. Dehydrate at 110 °C for 10 min.
3. Spin-coat with poly(dimethylsiloxane) (PDMS, Sylgard 184, pre-polymer : curing agent = 10:1, by weight, 500 rpm / 6 s, 3000 rpm / 30 s) and soft-bake at 110 °C for 25 seconds.
4. Laminate a flexible double side copper (Cu) coated polyimide (PI) (18 μm Cu / 25 μm PI / 18 μm Cu, DuPont) substrate on the glass and post-bake at 110 °C for 10 min.
5. Clean the double side copper (Cu) coated polyimide (PI) film with acetone, isopropyl alcohol (IPA), deinoized (DI) water and dehydrate at 110 °C for 10 min.
6. Spin coat 10 µm thick polyimide (YDPI-102, YiDun New Material Suzhou), baked at 250 °C for more than 2 hours.
7. Spin coat negative photoresist (PR) (AZ nLOF 2070, 500 rpm / 5 s, 3000 rpm / 30 s) and soft-bake at 110 °C for 2 min.
8. Expose with 365 nm optical lithography with irradiance for 45 mJ/cm^2^ (URE-2000/25, IOE CAS) through a chrome mask and post-exposure bake at 110 °C for 90s.
9. Develop PR in aqueous base developer (AZ300 MIF) and rinse with DI water.
10. Deposit 10 nm / 100 nm of Cr/Al as the markers by sputter coater.
11. Lift-off PR in acetone.
12. Clean the processed substrate in step 18 (acetone, IPA, DI water).
13. Dehydrate at 110 °C for 10 min.
14. Spin-coat with adhesive liquid (3000 rpm, 30 s) on the substrate and soft-bake at 110 °C for 4 min.
15. Transfer printing the LED from the source wafer onto the processed substrate with PDMS stamp.
16. Cure under to ultraviolet (UV) for 1 h and bake at 110 °C for 1 h.

*Epoxy encapsulation (LED)*

1. Clean the processed wafer in step 23 (acetone, IPA, DI water) and dehydrate at 110 °C for 10 min.
2. Expose to ultraviolet induced ozone (UV Ozone) for 10 min.
3. Spin-coat with epoxy SU8-2002 (500 rpm/ 5 s, 3000 rpm/ 30 s).
4. Soft-bake at 65 °C for 1 min and 95 °C for 1 min.
5. Pattern epoxy to reveal contact pads of LED by exposing with UV lithography with irradiance for 100 mJ/cm^2^ through a chrome mask.
6. Post-bake at 65 °C for 1 min and 95 °C for 2 min.
7. Develop in propylene glycol monomethyl ether acetate (PGMEA) for 1 min and rinse with IPA.
8. Hard bake at 110 °C for 20 min.

*LED interconnect metallization*

1. Clean the processed wafer in step 31 (acetone, IPA, DI water) and dehydrate at 110 °C for 10 min.
2. Pattern PR AZ nLOF 2070.
3. Deposit 10 nm / 600 nm / 200 nm of Cr/Cu/Au by sputter coater.
4. Lift-off PR in acetone.

*LED encapsulation*

1. Clean the processed wafer in step 35 (acetone, IPA, DI water) and dehydrate at 110 °C for 10 min.
2. Expose to ultraviolet induced ozone (UV Ozone) for 10 min.
3. Spin-coat with epoxy SU8-3005 (500 rpm/ 5 s, 3000 rpm/ 30 s).
4. Soft-bake at 65 °C for 1 min and 95 °C for 2 min.
5. Pattern epoxy by exposing with UV lithography tools with irradiance for 150 mJ/cm^2^ through a chrome mask.
6. Post-bake at 65 °C for 1 min and 95 °C for 3 min.
7. Develop in propylene glycol monomethyl ether acetate (PGMEA) for 1 min and rinse with IPA.
8. Hard-bake at 110 °C for 20 min.

*Diamond transfer printing*

1. Clean the processed wafer in step 43 (acetone, IPA, DI water) and dehydrate at 110 °C for 10 min.
2. Spin-coat with SU8-2002 (500 rpm/ 5 s, 3000 rpm/ 30 s).
3. Transfer printing the diamond from the source wafer onto the processed substrate with PDMS stamp.
4. Cure under to UV for 30 min and bake at 110 °C for 30 min.

*PEDOT:PSS interconnect metallization*

1. Clean the processed wafer in step 47 (acetone, IPA, DI water) and dehydrate at 110 °C for 10 min.
2. Spin-coat with SU8-3005 (500 rpm/ 5 s, 3000 rpm/ 30 s) and soft-bake at 65 °C for 1 min and 95 °C for 2 min.
3. Thicken the SU8-3005 by a second spin-coat process.
4. Pattern PR SU8-3005 (expose diamond) to buffer the height difference caused by diamond thickness, and hard-bake at 110 °C for 20min.
5. Clean the processed wafer in step 51 (acetone, IPA, DI water) and dehydrate at 110 °C for 10 min.
6. Pattern PR AZ nLOF 2070.
7. Roughen surface by reactive ion etching (RIE) with oxygen gas (O_2_, 100 sccm, 90 mTorr, 150 W) for 30s.
8. Deposit 500 nm of Au by sputter coater.
9. Lift-off PR in acetone.

*PEDOT:PSS coating*

1. Clean the processed wafer in step 56 (acetone, IPA, DI water) and dehydrate at 110 °C for 10 min.
2. Expose to ultraviolet induced ozone (UV Ozone) for 10 min.
3. Spin-coat with PEDOT:PSS (500 rpm/ 5 s, 2000 rpm/ 30 s) and bake at 110 °C for 1 h.
4. Clean the processed wafer in step 59 (acetone, IPA, DI water) and dehydrate at 110 °C for 10 min.
5. Spin coat positive PR (SPR220-3.0, Microchem, 500 rpm / 5 s, 3000 rpm / 30 s) and soft-bake at 110 °C for 1.5 min.
6. Pattern PR SPR220-3.0 by exposing with UV lithography tools with irradiance for 300 mJ/cm^2^ through a chrome mask and post-bake at 110 °C for 1.5 min.
7. Develop PR in aqueous base developer (AZ300 MIF), rinse with DI water and hard-bake at 110 °C for 10 min.
8. Clean PEDOT:PSS without PR covering by RIE with oxygen gas (O_2_ 100 sccm, SF_6_ 5 sccm, 90 mTorr, 150 W) for 2 min.
9. Lift-off PR in acetone.

*Encapsulation and laser milling*

1. Clean the processed wafer in step 65 (acetone, IPA, DI water) and dehydrate at 110 °C for 10 min.
2. Expose to ultraviolet induced ozone (UVO) for 10 min.
3. Pattern epoxy SU8 3005 (500 rpm/ 5 s, 3000 rpm/ 30 s), cure under to UV for 150 mJ/cm^2^ and bake at 100 °C for 30 min.
4. A bilayer of SU8 3005 by a second spin-coat process.
5. UV laser milling to form the probe shape, release from the glass substrates.
